# Advantages of Hydrogel-Based 3D-Printed Enzyme Reactors and Their Limitations for Biocatalysis

**DOI:** 10.3389/fbioe.2018.00211

**Published:** 2019-01-14

**Authors:** Barbara Schmieg, Johannes Döbber, Frank Kirschhöfer, Martina Pohl, Matthias Franzreb

**Affiliations:** ^1^Karlsruhe Institute of Technology, Institute of Functional Interfaces, Karlsruhe, Germany; ^2^Forschungszentrum Jülich GmbH, IBG-1: Biotechnology, Jülich, Germany

**Keywords:** enzyme immobilization, hydrogel, 3D-printing, mass transfer limitation, effectiveness factor, continuous flow reactor

## Abstract

The development of process steps catalyzed by immobilized enzymes usually encompasses the screening of enzyme variants, as well as the optimization of immobilization protocols and process parameters. Direct immobilization of biocatalysts by physical entrapment into hydrogels can be applied to reduce the effort required for immobilization, as the enzyme-specific optimization of the immobilization procedure is omitted. Physical entrapment is applicable for purified enzymes as well as crude cell extracts. Therefore, it can be used to quickly assess and compare activities of immobilized enzymes. For the application in flow reactors, we developed 3D-printed hydrogel lattices for enzyme entrapment as well as matching housings, also manufactured by 3D-printing. Testing the resulting enzyme reactors for three different enzymes, namely alcohol dehydrogenase from *Lactobacillus brevis*, benzoylformate decarboxylase from *Pseudomonas putida* and β-galactosidase from *Aspergillus oryzae*, and four different enzymatic reactions showed the broad applicability of the approach but also its limitations. The activity of the immobilized biocatalysts was measured in batch experiments and compared to the kinetics of the respective free enzymes in solution. This comparison yields an effectiveness factor, which is a key figure to describe the extent the immobilized catalyst is effectively utilized. For the examined systems the effectiveness factor ranged between 6 and 14% and decreased with increasing absolute activity of the entrapped enzymes due to mass transfer limitations. To test the suitability of the hydrogel lattices for continuous operation, they were inserted into 3D-printed reactor housings and operated at constant flow. Stable product formation could be monitored over a period of 72 h for all four enzymatic systems, including two reactions with redox cofactor regeneration. Comparing calculated and experimental conversion in the continuous setup, higher values of the effectiveness factor in batch experiments also hint at good performance in continuous flow. This can be used to optimize complex biocatalytic reactions on a small scale.

## Introduction

For the development of “green processes” on an industrial scale, there is a growing trend for continuous processes rather than batch reactions. Continuous process units are designed smaller and online-control of the conversion can be implemented to increase process robustness. Economically, productivity is increased and the amount of waste is reduced (Kamble and Yadav, 2017; Rao et al., 2017; Sheldon and Woodley, 2018). In the case of biocatalytic reactions, enzyme immobilization within the system allows separation and recycling of the biocatalyst as well as its application in different reactor concepts, e.g., in form of a fixed bed which is perfused by the substrate solution. Common approaches for immobilization include the attachment to magnetic particles, membranes, or porous matrices. Often unspecific covalent coupling methods are applied in order to avoid enzyme leaching that occurs in case of immobilization by physical adsorption, concomitant with respective contamination of the product (Cao, 2005; Sheldon, 2007; Mohamad et al., 2015). However, covalent binding via epoxide bonds and/or crosslinking reagents requires case-to-case optimization of the coupling procedure which often results in a substantial loss of enzyme activity (DiCosimo et al., 2013; Sheldon and Woodley, 2018). Besides, selective site-specific covalent binding methods have been developed such as the HaloTag^TM^ (England et al., 2015; Döbber and Pohl, 2017). An alternative to covalent coupling is the physical entrapment of biocatalysts into a porous matrix. Entrapment lacks the high selectivity of fusion-tag based immobilization strategies and introduces additional mass transfer limitations (Sheldon and Woodley, 2018). However, physical entrapment is a fast and simple process (Weiser et al., 2017) which is applicable to different enzymes over a wide pH range without the need of individual optimization. In addition, the entrapment is a mild process for the enzymes, and steric hindrance resulting from fixed bonding of the enzyme to the matrix is avoided (Robinson, 2015). Last but not least, the immobilization into porous matrices can also be utilized with non-purified material, such as crude cell extract. This is beneficial, as preliminary experiments can be done fast and without further purification (Tufvesson et al., 2011).

Besides immobilization, the development of continuous enzymatic reaction steps or even complex enzymatic cascade operations requires the availability of flexible screening platforms mimicking the flow regime in lab scale (Deng et al., 2015; Fraas et al., 2017). The challenge is to provide detailed data while minimizing the amount of raw material and enzyme needed for experiments on a lab scale. In this regard, 3D-printed devices are ideal (Kazenwadel et al., 2016; Gutmann et al., 2017; Peris et al., 2017). A scaled-down model of the process setup characterizes flow properties, mass transfer limitations, and mixing behavior more realistically than stirred batch systems, as geometry and process parameters can be chosen with regard to scaling laws. Individualized functional parts of the desired size and geometry can be attached to standard laboratory systems, and online control for high throughput (Bettermann et al., 2018; Gelhausen et al., 2018). Therefore, we developed a combination of tailored inert housings and functional immobilized catalyst structures, both manufactured in-house by suitable 3D-printing techniques, in order to generate a universally usable platform for rapid testing of continuous flow enzymatic processes.

**Figure 1:**
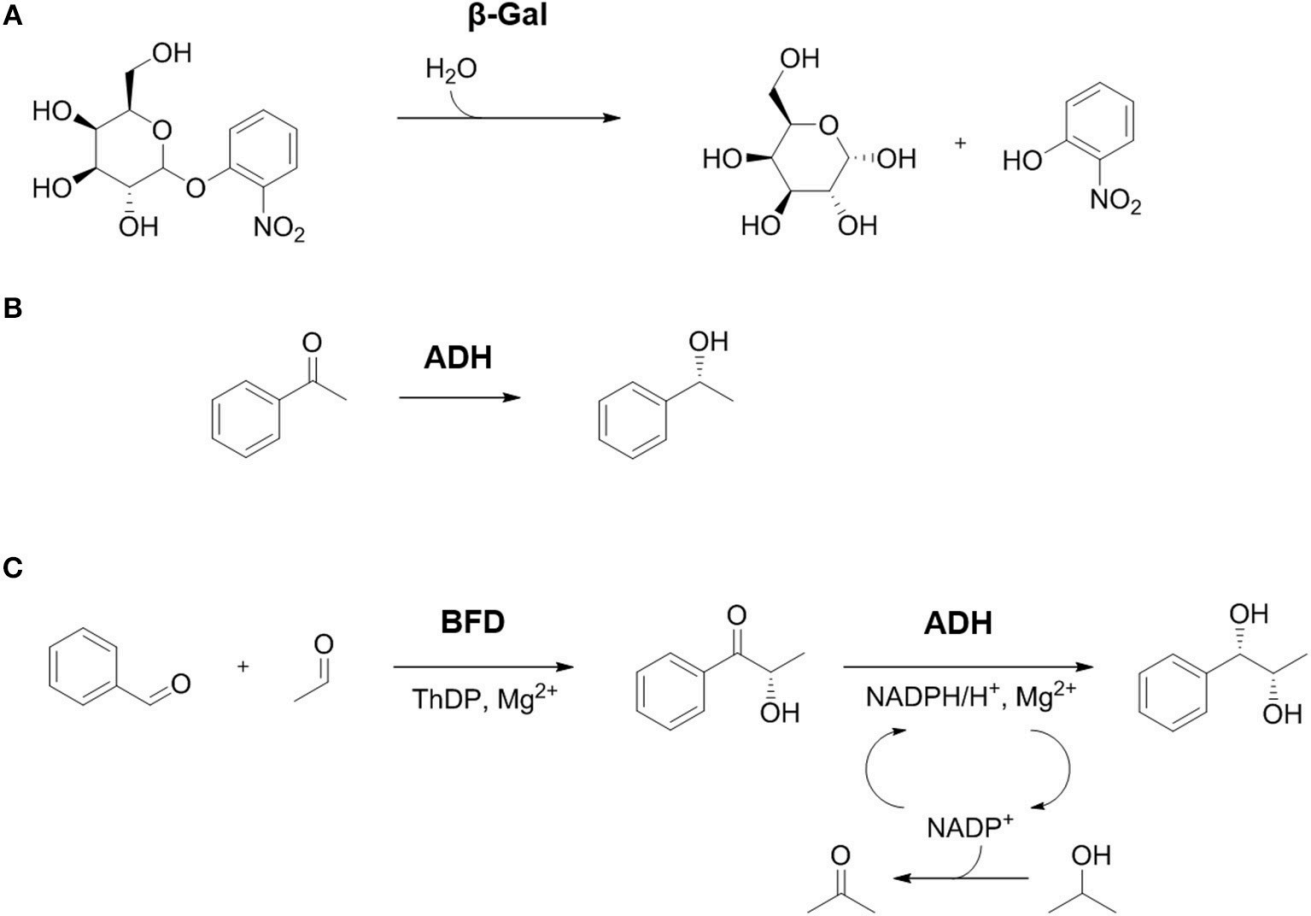
Overview of the studied reactions. **(A)** Hydrolysis of o-Nitrophenyl-β-d-galactopyranoside (ONPG) by β-Gal yields the monosaccharides galactose and o-nitrophenol. **(B)** Activity of ADH can be determined by enantioselective reduction of acetophenone to (*R*)-phenylethanol. **(C)** The combination of BFD and ADH in a 2-step cascade reaction. Carboligation of the educts benzaldehyde and acetaldehyde catalyzed by BFD yields the intermediate (*S*)-2-hydroxy-1-phenyl-propanone ((*S*)-HPP), which can be further reduced to the product (1*S*,2*S*)-1-phenylpropane-1,2-diol ((*S,S*)-PPD) by ADH. The redox equivalents are delivered by the cofactor NADPH that is oxidized to NADP^+^. For *in situ* regeneration of NADPH 2-propanol was added in excess which is oxidized to acetone by the same ADH.

To show the broad applicability of the platform, three different, physically entrapped enzymes were used. These three enzymes, β-galactosidase from *Aspergillus oryzae* (β-Gal), benzoylformate decarboxylase from *Pseudomonas putida* (BFD), and alcohol dehydrogenase from *Lactobacillus brevis* (ADH), were applied in four scenarios with increasing complexity (see Table 1 and Figure 1). The data resulting from the respective processes reveal valuable insights into reaction kinetics and mass transfer limitations of the processes as well as performance of the 3D-printed reactor setups. β-Gal is industrially used to produce lactose-free dairy products on a large scale (Mlichova and Rosenberg, 2006; Grosová et al., 2008; Robinson, 2015). Furthermore, the synthesis of tailored oligosaccharides for pharmaceutical or food industry is possible in technical setups with β-Gal (Brakowski et al., 2016). Its main substrate is the disaccharide lactose, which is cleaved into galactose and glucose (Zhang et al., 2016). Besides, also O-Nitrophenyl-β-d-galactopyranoside (ONPG) is hydrolyzed by the enzyme which enables the photometric detection of the yellow cleavage product o-nitrophenol (Figure 1A) under alkaline conditions (Miller, 1972). BFD catalyzes the decarboxylation of benzoylformate to benzaldehyde in the mandelate catabolism. As a site reaction, BFD also mediates the synthesis of chiral 2-hydroxy ketones such as (*S*)-HPP starting from benzaldehyde and acetaldehyde (Wilcocks and Ward, 1992; Iding et al., 2000) (Figure 1C). For optimal activity, the enzyme requires magnesium ions and thiamine diphosphate. ADH catalyzes oxidoreductions of a huge variety of ketones and alcohols, respectively (Leuchs and Greiner, 2011). It reveals high activity toward ketones with only one bulky side chain such as acetophenone and derivatives thereof (Rodríguez et al., 2014; Döbber et al., 2018b) as well as 2-hydroxy ketones like (*S*)-HPP (Kulig et al., 2012; Wachtmeister et al., 2016; Döbber et al., 2018a). NADPH is required as a reducing equivalent for the reaction. To minimize the consumption of this expensive cofactor, *in-situ* regeneration can be implemented. For example 2-propanol is oxidized to acetone, thereby providing the reducing equivalents for NADP^+^ to regenerate NADPH for the main enzymatic reaction (Leuchs and Greiner, 2011). The activity of ADH can be estimated with the commercially available substrate acetophenone, yielding (*R*)-phenylethanol (Figure 1B). In addition, BFD and ADH can be combined in a two-step enzymatic cascade. In the first step, BFD is applied for the production of (*S*)-HPP which is then reduced in a second step toward the target vicinal diol (*S,S*)-PPD (Figure 1C). This cascade was implemented in batch in aqueous solvent (Kihumbu et al., 2002) as well as in a micro-aqueous reaction system (Wachtmeister et al., 2016). Additionally, a continuous two step cascade in flow was recently successfully set up (Döbber et al., 2018a). The products of the reaction cascade, a diastereomeric diol, is of interest for the pharmaceutical industry, as it can be produced with high optical purity (Valinger et al., 2014; Porta et al., 2016).

**Table 1:** Properties of the applied enzymes and model reactions. The complexity of the investigated enzymatic reactions ranges from the simple case of cleaving the model substrate ONPG by the enzyme β-Gal up to the cofactor-dependent cascade reaction of BFD and ADH.

**Applied enzyme**	**β-Gal**	**BFD**	**ADH**	
Substrates	ONPG	Benzaldehyde Acetaldehyde	Acetophenone	(*S*)-HPP (cascade intermediate)
Products	O-nitrophenol Galactose	(*S*)-HPP	(*R*)-Phenylethanol	(*S,S*)-PPD
Cofactor	None	Magnesium Thiamine disphosphate (both added in flow)	NADPH (added in flow non-equimolar; regeneration in flow by 2-propanol)	
Mass of monomer [kDa]	105–106 monomeric enzyme (Tanaka et al., 1975; Maksimainen et al., 2013)	56 Quaternary structure tetramer (Hasson et al., 1998)	27 Quaternary structure tetramer (Niefind et al., 2003; Schlieben et al., 2005; Leuchs and Greiner, 2011)	
pH optimum	4.6 (Tanaka et al., 1975)	8 (Iding et al., 2000)	7 for reductions (9 for oxidations) (Riebel, 1996)	

## Materials

### Enzymes and Buffers

β-Gal (EC 3.2.1.23) from *A. oryzae* and the substrate ONPG were obtained from Sigma-Aldrich (Darmstadt, Germany) and used without further purification. Reaction buffer for experiments with β-Gal was sodium citrate (333 mM, pH 4.6) adjusted with NaOH (all obtained from VWR, Radnor, USA, unless specified differently). BFD from *P. putida* was produced in *Escherichia coli* BL21(DE3) according to Gocke et al. (2009). For the experiments one batch of freeze-dried crude cell extract was used. All experiments with BFD were carried out in potassium phosphate buffer (50 mM, pH 7), containing 2.5 mM MgSO_4_ and 0.15 mM thiamine diphosphate (purity >95%) as cofactors. The substrates benzaldehyde (purity >99%) and acetaldehyde were obtained from Sigma-Aldrich. Acetaldehyde was always used in 2.5-fold molar excess relative to the concentration of benzaldehyde. ADH from *L. brevis* was produced according to Kulishova et al. (2010), and used as freeze-dried crude cell extract. For the ADH-catalyzed reduction of acetophenone, 10% (v/v) 2-propanol and 0.5 mmol/l NADP^+^ (purity ≥97%) were added to the phosphate buffer, which was also used for the BFD system. For the ADH-catalyzed reduction of (*S*)-HPP, a product solution from the previous BFD reactor experiments was used, which contained about 10 mM (*S*)-HPP. Prior to the reaction, excess acetaldehyde in this solution was evaporated in a beaker under stirring at 35°C for 30 min and allowed to cool down to room temperature; before 2-propanol (10% v/v) as well as 0.5 mmol/l NADP were added. For all experiments, the enzymes were used with the named respective buffer systems.

### Hydrogel Preparation and 3D-Printing

For the immobilization of β-Gal, a hydrogel based on poly(ethylene glycol) diacrylate (PEG-DA; average M_n_ 700; Merck KGaA, Darmstadt, Germany) and the viscosity-enhancing material Laponite RD (LRD; BYK-Chemie GmbH, Wesel, Germany) was prepared for extrusion 3D-printing according to (Schmieg et al., 2018). Weight contents were as follows: 22.3% (w/v) PEG-DA, 2.2% (v/v) 2-hydroxy-4′-(2-hydroxyethoxy)-2-methylpropiophenone (Merck KGaA, Darmstadt, Germany) dissolved 100 g/l in 70% (v/v) ethanol in water and 2.5% (v/v) β-Gal solution (10 g/l) were mixed with a gel of LRD suspended in water (5.0% w/v). 3D-printing of hydrogel structures was done with a 3D-Discovery extrusion-based 3D-printer (regenHU, Villaz-St-Pierre, Switzerland) according to Schmieg et al. (2018). The size of the 3D-printed lattices was 13 × 13 × 3 mm3 with a strand distance of 1.5 mm.

In case of BFD and ADH, the 3D-printing system was used to print support structures with a grid width of 2 mm consisting of 5.0% (w/v) LRD in water. In a layer-by-layer process, each layer of support structure was printed, then manually filled with a liquid PEG-DA mixture, which contained the crude cell extract, and hardened by UV-light. Crude cell extract was mixed with the PEG-DA mixture which consisted of 1.0 ml buffer, 0.4 ml PEG-DA, 0.04 ml initiator solution [100 g/l 2-hydroxy-4′-(2-hydroxyethoxy)-2-methylpropiophenone dissolved in 70% (v/v) ethanol in water] and about 60 mg lyophilized crude cell extract of BFD, respectively ADH. The support material was dissolved by immersing the printed hydrogel into buffer at room temperature for 10 min, which also removes the negligible amount of leached enzymes (Schmieg et al., 2018).

### Activity Tests With Free Enzymes

Initial activities of the free enzymes were tested in batch in an Eppendorf Thermoshaker (Eppendorf, Hamburg, Germany) at 750 rpm for 20 min using discontinuous activity assays.

For β-Gal, reaction volumes of 1.8 ml with c_enzyme_ = 10 mg/l and c_substrate_ in the range of 0.07–8.9 mM were incubated at 37°C. Enzymatic activity was inactivated in collected samples of 25 μl with 100 μl 1M Na_2_CO_3_ solution pH 11.8. Citrate buffer was added up to a total volume of 200 μl (Brakowski et al., 2016) resulting in a solution with a pH of 9. The samples were analyzed photometrically at 420 nm in an Enspire 2300 Multimode plate reader (Perkin Elmer, Inc., Waltham, USA).

For measuring the activity of BFD, the parameters were 37°C, V = 1.6 ml, c_extract_ = 1 g/l, and c_benzaldehyde_ = 5–35 mM with acetaldehyde in 2.5-fold excess in potassium phosphate buffer. To stop the enzymatic reaction, samples of 20 μl were added to 480 μl of a mixture of acetonitrile with toluene as internal standard as previously described (Döbber et al., 2018b). After centrifugation (10,000 rpm, 4 min, centrifuge type 5415R, Eppendorf, Hamburg, Germany) to remove particles samples were analyzed with a 1100 Series HPLC system (Agilent, Santa Clara, USA) equipped with a Chiralpak IE column (Chiral Technologies Europe SAS, France) and a DAD detector (wavelengths 190–400 nm). Isocratic elution was performed with 50%vol acetonitrile in water at 20°C with a flow of 1 ml/min (Döbber et al., 2018a).

ADH experiments were done with c_extract_ = 1 g/l at a temperature of T = 25°C. For the reaction with the acetophenone, substrate concentrations between 6 and 44 mM were used. The concentration of (*S*)-HPP was in the range of 2.7–9.6 mM. Samples (20 μl) were taken, added to 480 μl of a mixture of acetonitrile with toluene as internal standard and analyzed with HPLC analog to samples of BFD experiments.

The data of the determined volumetric activities *v* were fitted to the Michaelis–Menten kinetic model using the MATLAB R2017a Curve-Fitting tool (Mathworks Inc., Natick, USA). Fit options were: Nonlinear Least Squares, Robust: off, Algorithm: Trust-Region, number of iterations: 1,000, coefficients K_i_, v_max_ ϵ (0; infinity), 95% confidence interval. First order Michaelis–Menten kinetics (Deichmann et al., 2014) was applied for β-Gal and ADH (Equation 1), for the two-substrate reaction of BFD, the volumetric activity was fitted by applying second order kinetics (Equation 2) (Laidler and Velick, 1983).

(1) $$\begin{array}{l} v_{reaction}=\;\frac{v_{\max}*\left[A\right]}{K_{m}+\left[A\right]} \end{array}$$

(2) $$\begin{array}{l} v_{reaction}=\;\frac{v_{\max}*\left[A\right]*\left[B\right]}{K_{m,A}*K_{m,B}\;+\;{\left[A\right]*K}_{m,B}\;+\;{\left[B\right]*K}_{m,A}\;+\;\left[A\right]*\left[B\right]} \end{array}$$

### Activity Tests With Entrapped Enzymes

Activity tests with β-Gal entrapped in hydrogel were conducted with lattice structures of about 400 mg in a total volume of 6 ml educt solution (c_enzyme_ = 10 g/l in the stock solution for preparing the hydrogels) according to Schmieg et al. (2018). Hydrogel structures of 280 mg each containing BFD and ADH were prepared manually to avoid the use of support material. For this, the liquid PEG-DA solution with lyophilized enzyme crude extract (see section Hydrogel Preparation and 3D-Printing) was pipetted into silicone formwork discs (diameter of 12 mm) and hardened with UV-light for 10 min (VL-8.L lamp, Vilber Lourmat, Marne-la-Vallée, France). The hydrogel discs were immersed in buffer for 10 min to remove unbound material. Afterwards, they were cut into three pieces with a scalpel and added to 1.6 ml of the respective substrate solution. To calculate the increase of product in solution against time a linear regression using the MATLAB Curve-Fitting tool was used. Fit options were: Nonlinear Least Squares, Robust: off, Algorithm: Trust-Region, number of iterations: 1,000, coefficients K_i_, v_max_ ϵ (0; infinity), 95% confidence interval. To approximate the initial velocity of the reaction, data points between 0 and 20 min were taken into account.

### Reactor Tests

**Figure 2:**
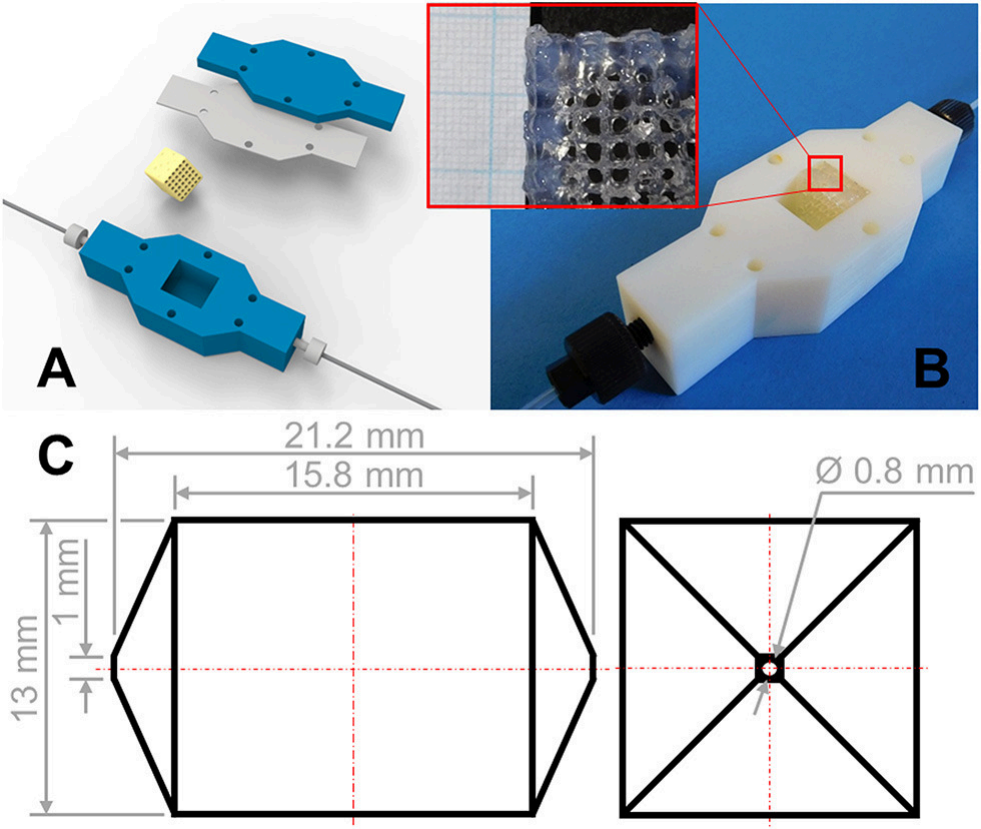
**(A)** Isometric view of the reactor assembly. The hydrogel structure is inserted in the reactor housing with connection to the fluidic system. The lattices are placed into the reactor with the plane of the highest porosity perpendicular to the flow direction. The assembly is closed with a sealing and lid. **(B)** Experimental view of the reactor configuration. The reactor chamber is not completely filled with hydrogel lattices, which are shown in the detail picture. **(C)** Dimensions of the 3 ml reactor chamber.

Reactor experiments were done with a 3D-printed reactor chamber (see Figure 2) with a square cross-section of 13 × 13 mm2, pyramid-shaped inlet and outlet and a total chamber volume of 3 ml. For the 3D-printing of the housing, the commercially available 3D-printing system Objet Eden260V equipped with VeroWhitePlus RGD835 material (both Stratasys, Eden Prairie, USA) was used as described earlier (Kazenwadel et al., 2016). The reactor was connected to the fluidic system by coned PEEK fittings with 10–32 thread (IDEX Health & Science, LLC, Oak Harbor, USA). At the beginning of an experiment, the reactor system and the fluidic channels (teflon tubes, inner diameter of 0.8 mm, outer diameter 1.6 mm, VWR, Radnor, USA) were filled with buffer to dispel residual air bubbles. Four hydrogel lattice structures (printed as described in section Hydrogel Preparation and 3D-Printing) were placed within the reactor chamber (average mass of about 1.5 g hydrogel). Because of slight swelling of the hydrogel within the buffer solution its edges get in close contact to the walls of the reactor chamber, minimizing undesired channeling flows. Buffer was added to expel air before the reactor was closed with an in-house manufactured flat gasket (material: silicone 40° shore A, thickness 1 mm, Exact plastics, Bröckel, Germany) and a lid. Substrate solution was pumped through the system with flowrates of 1–3 ml/h by a syringe pump (Pump 11, Harvard Apparatus, Holliston, USA). Product solution was collected in fractions of 1 ml in a fraction collector (Superfrac, Pharmacia Biotech AB, Uppsala, Sweden) and analyzed afterwards. Temperature was controlled by immersing the reactor into a water bath (Lauda E 100, Lauda-Königshofen, Germany).

## Results

### Michaelis–Menten Kinetics in Solution

**Figure 3:**
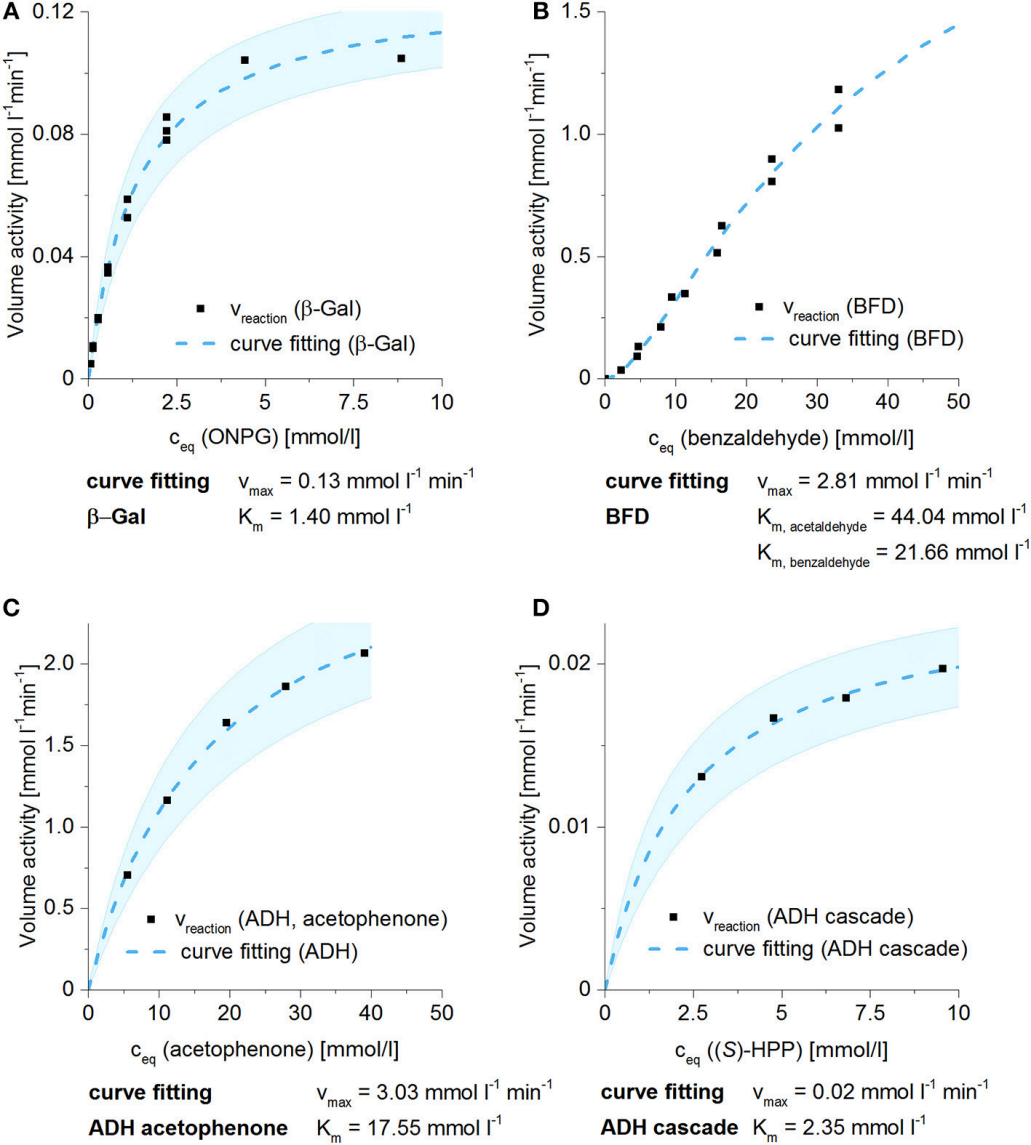
Michaelis–Menten kinetics of the investigated biocatalytic reactions with freely dissolved enzymes. Kinetic parameters v_max_ and K_m_ were calculated by fitting the data to the Michaelis–Menten function, which is shown including 95% confidence bounds. In the whole figure, each data point represents one sample. **(A)** β-Gal kinetics for the cleavage of ONPG at pH 4.6 (*n* = 3 separate runs). **(B)** BFD kinetics for the carboligation of benzaldehyde and a respective 2.5-fold excess of acetaldehyde (*n* = 2 separate runs). Estimation of the confidence bounds was omitted because of limited data for substrate excess. **(C)** ADH kinetics for the reduction of acetophenone. **(D)** ADH kinetics for the reduction of (*S*)-HPP, which was previously synthesized by BFD. For ADH kinetics, data points were generated in one batch due to shortage of the enzyme.

In order to evaluate the suitability of 3D-printed enzyme immobilizates for biocatalytical applications, the properties of the respective freely dissolved enzymes, shortly named free enzymes in the following, were determined as a reference (see Table 2 and Figure 3). Especially the reaction kinetics depending on the actual substrate concentration is of interest, because the actual substrate concentration in the hydrogel immobilizates is assumed to be lower compared to the substrate solution due to mass transfer limitations. On the other hand, formed products may accumulate in the hydrogel and their concentration in solution could be therefore be lower. Therefore, volumetric activities vs. substrate concentration were determined for all used enzyme/substrate combinations (see Figure 3). ONPG, the substrate used for the β-Gal experiments, is soluble up to concentrations of 13.3 mM. The purified enzyme with first order reaction kinetics has a K_m_ value of 1.40 mM and v_max_ of 0.13 mmol l^−1^ min^−1^. Comparing K_m_ with the maximum solubility of ONPG shows that maximum reaction velocity is easily reached by applying ONPG concentrations >5 mM.

**Table 2:** Overview of the used parameters for enzyme kinetics and the calculated kinetic parameters for experiments in solution and hydrogel lattices.

**Applied enzyme**		**β-Gal**	**BFD**	**ADH aceto phenone**	**ADH cascade**
**FREELY DISSOLVED ENZYMES**					
c_enzyme/crudecellextract, solution_	g l^−1^	0.01	1	1	1
c_substrate_	mmol l^−1^	2.2	35	39	4.8
v_reaction, solution_	mmol l^−1^min^−1^	0.08	1.15	2.09	0.016
k_app, solution, experimental_	min^−1^	0.036	0.033	0.053	0.0034
v_max_ (with 95% confidence bounds)	mmol l^−1^min^−1^	0.13 (0.12, 0.14)	2.81	3.03 (2.77, 3.28)	0.02 (0.02, 0.03)
K_m_ (with 95% confidence bounds)	mmol l^−1^	1.40 (1.12, 1.68)	K_m, acetaldehyde_ 44.04	17.55 (14.09, 21.01)	2.35 (1.75, 2.95)
			K_m, benzaldehyde_ 21.66		
**ENTRAPPED ENZYMES**					
c_enzyme, hydrogel_ referred to V_solution_	g l^−1^	0.011	6	4.4	4.4
c_enzyme, hydrogel_ referred to V_hydrogel_	g l^−1^	0.25	40.7	26.2	34.9
c_substrate_	mmol l^−1^	2.2	35	44	4.8
V_solution_	ml	6	4.1	1.7	1.6
V_hydrogel_	ml	0.26	0.6	0.28	0.2
v_reaction, hydrogel_ referred to V_solution_	mmol l^−1^min^−1^	0.006	0.41	0.91	0.01
v_reaction, hydrogel_ referred to V_hydrogel_	mmol l^−1^min^−1^	0.137	2.821	5.470	0.080
k_app, hydrogel, experimental_	min^−1^	0.062	0.081	0.124	0.017
k_app, hydrogel, theoretical_	min^−1^	0.894	1.343	1.403	0.120
Effectiveness factor η	%	6.9	6.0	8.9	14.0

Second order reaction kinetics was applied to describe the reaction of benzaldehyde and acetaldehyde catalyzed by the enzyme BFD (Iding et al., 2000) as the two reaction partners acetaldehyde and benzaldehyde have to be in spatial proximity in the enzyme active site to react. The experimental data with an increasing slope with benzaldehyde concentrations lower than 10 mM is in accordance with this model. At benzaldehyde concentrations of 40 mM, which is the solubility limit at room temperature (22°C) in the applied buffer, the plateau of the Michaelis–Menten curve indicating v_max_ of the enzyme is not reached. K_m_,_B_ of 21.7 mM for benzaldehyde and K_m_,_A_ of 44.0 mM for acetaldehyde were determined for the BFD crude cell extract under these experimental conditions. The confidence interval gets very broad because of the limited amount of data for substrate excess and is therefore not applied.

The ADH crude extract showed a K_m_ of 18 mM for the substrate acetophenone. Thus, reaction rates with almost maximum velocity can be reached in aqueous solution at room temperature, applying substrate concentrations close to the maximum solubility of acetophenone in water (about 57 mM). Compared to acetophenone, the activity of ADH for the substrate (*S*)-HPP is about 10-fold less (Figures 3C,D). Applying the acetaldehyde-free product solution obtained by BFD as a substrate solution for ADH reactions resulted in a v_max_ value of 0.02 mmol l^−1^ min^−1^ and a K_m_ value of 2.4 mM. The volumetric activity of ADH for (*S*)-HPP is also about 10 times slower than BFD activity for benzaldehyde. When BFD and ADH are combined in a cascade, either the amount of applied enzymes or the residence times have to be adjusted to optimize the overall yield.

In summary, the volumetric activities for the chosen enzyme/substrate systems span a range of about two orders of magnitude, when substrate concentrations close to the respective maximum solubility were applied. Under the experimental conditions used, β-Gal reaction velocity approached v_max_, whereas the activity of BFD is almost linear dependent on the available substrate concentration. Therefore, the model systems cover an interesting range of biocatalytic conditions, which allow comprehensive insight into the effects of immobilization via enzyme entrapment into printed hydrogels.

### Effective Activity of Enzymes Entrapped in Hydrogels

**Figure 4:**
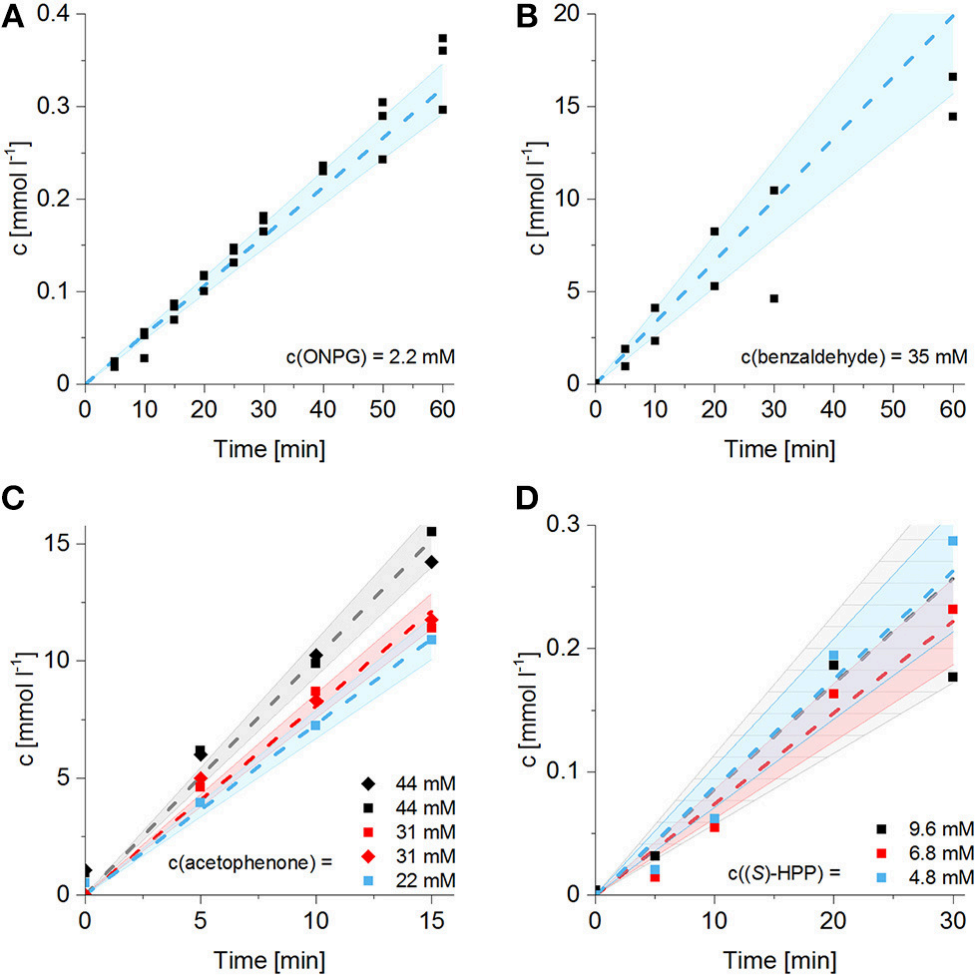
Conversion curves in batch with enzymes entrapped in hydrogel lattices by following the formation of the respective product over time. **(A)** β-Gal kinetics for the cleavage of 2.2 mM ONPG at pH 4.6 (*n* = 3 separate runs). **(B)** Formation of (*S*)-HPP by BFD starting from 35 mM benzaldehyde and 87.5 mM acetaldehyde (*n* = 2 separate runs). **(C)** ADH-catalyzed reduction of three different concentrations of acetophenone (*n* = 2 separate runs). **(D)** ADH-catalyzed reduction of three different concentrations of (*S*)-HPP, provided by BFD reactor experiments (*n* = 1). Linear regression with a 95% confidence interval of the slope was applied.

For the determination of the initial enzyme activity, the enzymes were entrapped into crosslinked hydrogels, as described in section Activity Tests With Entrapped Enzymes. Afterwards, the hydrogel lattices were transferred into small batch reaction vials filled with the respective substrate solutions and the amount of the product formation over time was determined (see Figure 4). For β-Gal and BFD, the experiment was conducted in triplicate or duplicate. In the case of ADH, different initial substrate concentrations were tested for acetophenone and (*S*)-HPP. In the case of acetophenone, the concentrations were chosen in a range between first reaction order and reaction order zero of the respective Michaelis–Menten kinetics of free enzymes, whereas for (*S*)-HPP the highest chosen concentrations (9.6 mM) was close to the substrate saturation range of the soluble enzyme. As demonstrated in Figures 4C,D the volumetric activities of ADH follow the expectations, with a moderate substrate concentration dependence in the case of acetophenone and almost no substrate concentration dependence in the case of (*S*)-HPP. In all cases, the almost linear progression of product formation shows that the applied amount of substrate is not exhausted in the observed reaction times and that no product inhibition occurs. The slopes of the curves in Figure 4 correspond to the effective volumetric activities of the systems with enzymes entrapped in hydrogels. Nevertheless, comparison of the effective volumetric activities of entrapped enzymes with those of the corresponding systems including free enzyme shows that entrapment reduces the effective activity. In the following section, the degree of this reduction will be investigated in more detail.

### Comparison of Enzyme Activity in Solution and Entrapped in Hydrogel

From classical heterogeneous catalysis with porous carriers it is known that the effectiveness of the entrapped catalyst depends on mass transfer limitations induced by properties of the carrier, the diffusion coefficient of the substrate, but also the reaction rate of the catalytic reaction. For the crude cell extracts of ADH and BFD but also in case of the dextrin-stabilized β-Gal preparation, the applied enzyme concentration and therefore the turnover number of the enzyme is not known. A first approximation of the expected activities and the activity reduction can be derived by treating the reactions as first order reactions with an apparent reaction rate constant (*k*_*app*_).

(3) $$\begin{array}{l} v_{reaction}=\;k_{app}\cdot c_{substrate} \end{array}$$

Because volumetric activities also depend on the applied enzyme concentration, one has to keep in mind, that *k*_*app*_ is a function of the local enzyme concentration. Therefore, assuming that the intrinsic enzyme activity is not affected by the entrapment in the hydrogel, the theoretical apparent reaction rate constant within the hydrogel can be calculated by:

(4) $$\begin{array}{l} k_{app,hydrogel,theoretical}=\;k_{app,solution}\;\cdot \;\frac{c_{enzyme,hydrogel}}{c_{enzyme,solution}} \end{array}$$

**Figure 5:**
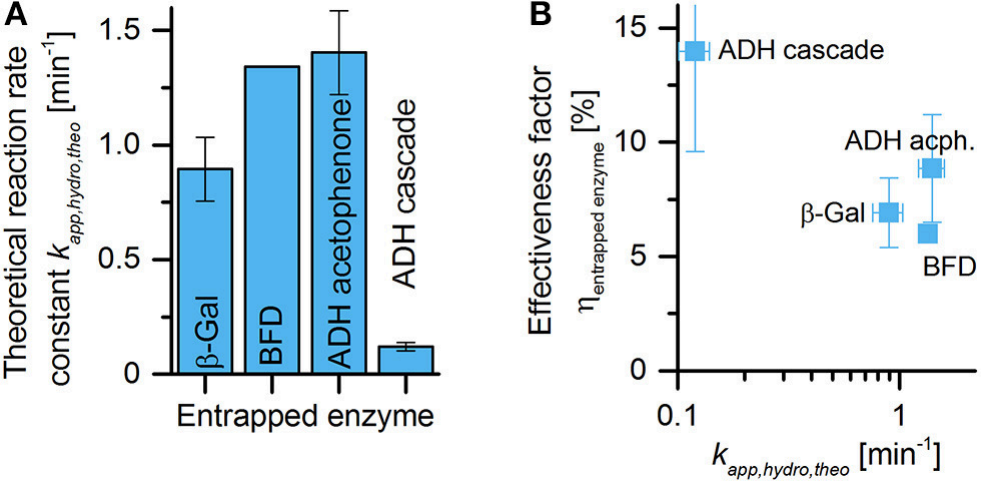
Comparison of enzyme activities in solution and in batch experiments with submersed hydrogel structures. **(A)** Local volumetric activities of the used enzyme/substrate systems within the hydrogel. **(B)** Calculated ratios between the effective volumetric activities of entrapped enzymes and the volumetric activities of the corresponding free enzymes, plotted vs. the local volumetric activities of the enzymes in the hydrogel.

Table 2 lists the apparent reaction rate constants calculated from the measured volumetric activities in batch experiments with freely dissolved enzyme. It also lists the enzyme concentrations in the hydrogels for the experiments with entrapped enzyme and the resulting theoretical apparent reaction rate constants within the hydrogels, calculated by Equation (4). As can be seen from Figure 5A, these theoretical apparent reaction rate constants differ by about an order of magnitude, with the value for the ADH-catalyzed reduction of (*S*)-HPP being between seven to 12 times lower compared to both other enzymatic reactions investigated in this study. It can be expected that high reaction rate constants within the hydrogel enhance mass transfer limitations for both, the respective substrates and the products. This causes a reduction of the effective volumetric activity of entrapped enzymes compared to their free form. This hypothesis is tested in Figure 5B, which shows the effectiveness factors of the enzymes entrapped in hydrogel plotted vs. the theoretical apparent reaction rate constants. The effectiveness factor is defined as the ratio between the experimentally observed and the theoretical reaction rate within the hydrogel.

(5) $$\begin{array}{l} \eta =\;\frac{v_{reaction,\;hydrogel,\;experimental}}{v_{reaction,\;hydrogel,theoretical\;}} \end{array}$$

Applying Equations (3) and (4), Equation (5) converts into:

(6) $$\begin{array}{l} \eta =\frac{k_{app,\;hydrogel,\;experimental}}{k_{app,\;hydrogel,theoretical\;}}\\ \;=\frac{k_{app,\;hydrogel,\;experimental}}{k_{app,\;solution\;}}\;\cdot \;\frac{c_{enzyme,\;solution}}{c_{\;enzyme,hydrogel}} \end{array}$$

Although there is some variation of the effectiveness factor at higher theoretical reaction rate constants, Figure 5B clearly shows the expected relationship. If the same hydrogel preparation is used, systems with higher theoretical reaction rate constants result in reduced effectiveness factors due to increased mass transfer limitations. While the reduction of (*S*)-HPP by ADH with a theoretical reaction rate constant of around 0.1 min^−1^ results in an effectiveness factor of at least 14%, the other reactions with theoretical reaction rate constants of around 1 min^−1^ result in effectiveness factors only in the range of 6–9%.

### Enzyme Activity in Flow Reactors

**Figure 6:**
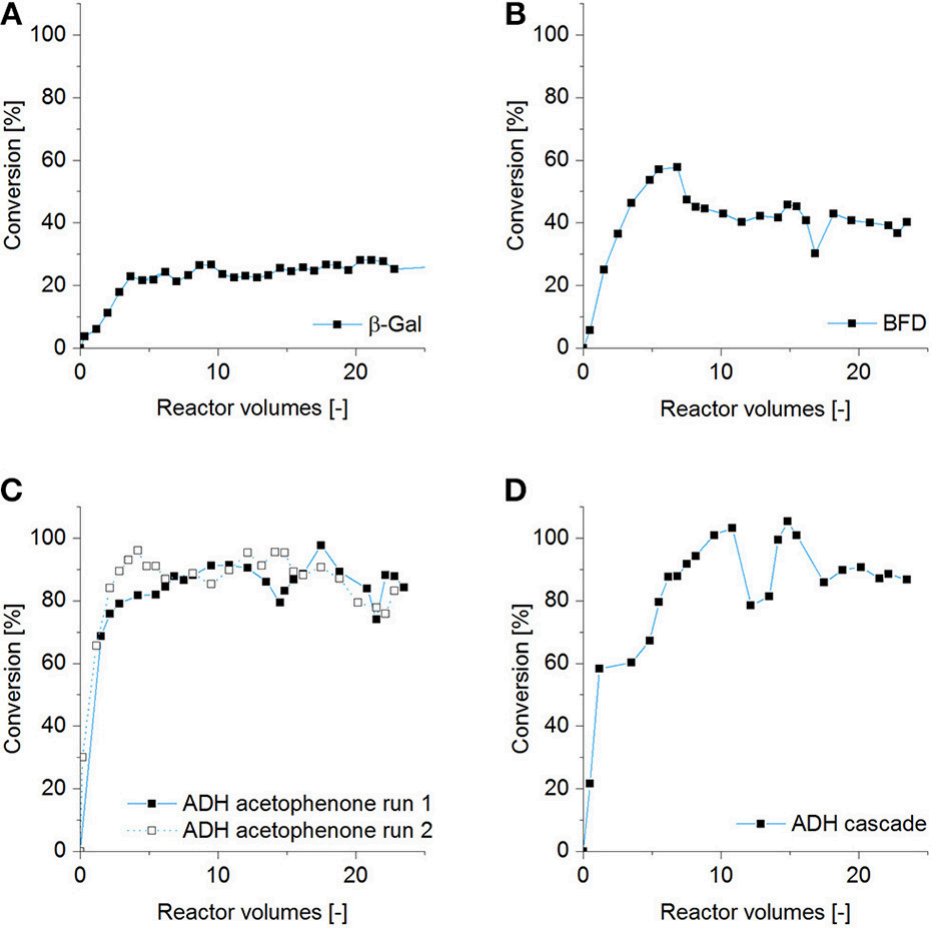
Detected conversion to the respective product compound at the exit of the 3 ml reactor system of one exemplary run. Parameters were **(A)** β-Gal (*m* = 0.38 mg/reactor) with ONPG substrate 2.2 mM, flow rate 0.05 ml/min. **(B)** BFD (*m* = 56 mg/reactor) catalyzing the carboligation of benzaldehyde (25 mM) and acetaldehyde (62.5 mM), flow rate 0.017 ml/min. **(C)** ADH (*m* = 42 mg/reactor) reducing acetophenone (50 mM), flow rate 0.017 ml/min. Reproducibility is shown by a second experiment with identical parameters. **(D)** ADH (*m* = 58 mg/reactor) reducing (*S*)-HPP provided by BFD reactor experiments (11 mM), flow rate 0.017 ml/min.

To exploit effects which happen by switching the mode of operation from batch to continuous, we inserted directly or indirectly 3D-printed porous hydrogel structures with entrapped enzymes into a rigid 3D-printed housing in order to quickly assemble a flow reactor which allows continuous biocatalytic reactions (Figure 2). Neglecting the inlet and outlet zones, the central part of the reaction chamber has a volume of about 2.5 ml of which around 1.5 ml were filled by hydrogel and 1 ml was formed by the flow channels through the hydrogel insert. The flow reactors were operated between 1 and 3 days as described in section Reactor Tests. During this time feed volumes corresponding to more than 24 reactor volumes were pumped through the reactors. After a start-up period, the conversion curves show stable operation for all three immobilized enzymes over the course of 20 reactor volumes (see Figure 6). Steady product concentrations were reached after a flow of about 5 reactor volumes. In the case of β-Gal also longer operation times of up to 10 days (corresponding to more than 200 reactor volumes), including storage at 5°C over the weekend, have been successfully tested (data not shown). This indicates the applicability of the hydrogel system for enzyme immobilization over longer periods of time, which is in accordance with β-Gal storage experiments shown by the group of J. Hubbuch (Radtke et al., 2017) with a comparable PEG-DA material.

In the case of β-Gal the applied flow rate was 0.05 ml/min (3 ml/h). Taking into account the open channel volume in the hydrogel of around 1 ml, this shows that the average residence time of the fluid in the hydrogel insert is around 20 min. As can be seen in Figure 6, a conversion of only 25–30% was observed. A relatively low conversion yield of about 40% was also determined for the BFD system, although the flow rate was decreased to 0.017 ml/min (1 ml/h). The conversion is determined by the measured concentration of the product (*S*)-HPP in the effluent. However, preliminary tests showed that the educt benzaldehyde is adsorbed by the polyacrylate material of the reactor housing as well as within the PEG-DA hydrogel and thus not available for the enzymatic reaction. In case of the two biocatalytic systems based on ADH high conversions yields of around 90% could be reached, although we know from the free enzyme in solution and from the batch hydrogel experiments that the volumetric activity of ADH for conversion of the substrate acetophenone is almost ten times higher compared to (*S*)-HPP. In the following, the interrelation between enzyme activities determined in batch experiments with hydrogel lattices and the performance of printed enzyme reactors with continuous flow will be discussed.

### Comparison of the Activity of Entrapped Enzymes in Batch and Flow Reactors

**Figure 7:**
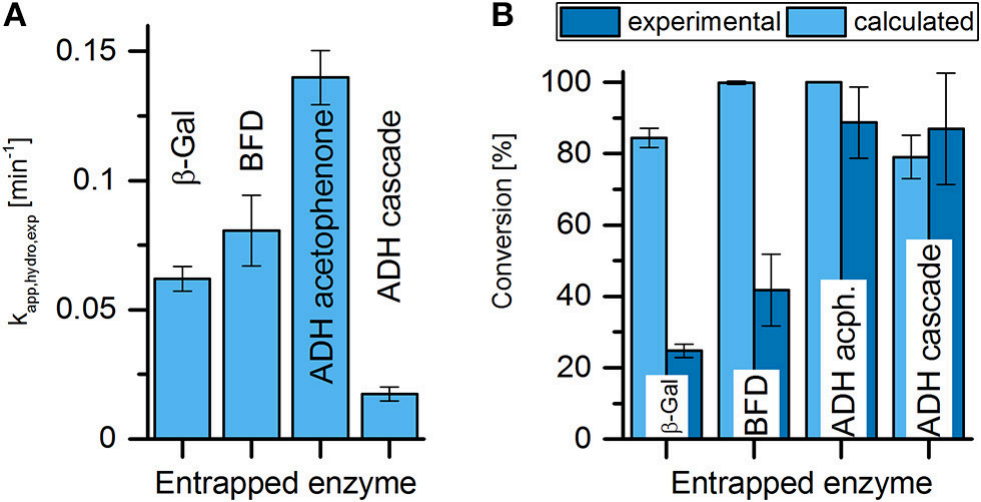
Comparison of expected and experimental conversion in flow reactor experiments. **(A)** Reaction rate constant calculated from batch experiments for the respective enzymes. **(B)** Expected conversion in the flow reactor experiments based on first-order kinetics, calculated with _kapp, hydro, exp_ **(A)** compared to the experimental conversion of the respective reactor experiments (see Figure 6) in the steady state.

As a starting point to estimate enzyme performance in a flow reactor, it is important to know how these results correspond to the results of the batch hydrogel tests. Thereby, the time and flow regimes of the systems have to be taken into account: in the initial phase of the batch experiments, which is used for the determination of reaction kinetics, the substrate concentration in solution is assumed to be practically constant. In contrast, even in the steady state of the flow reactor, the substrate concentration varies while the solution flows through the reactor. The easiest way to consider the influence of a decreasing substrate concentration is, again, to regard the conversion as an apparent first order reaction. The required experimental apparent reaction rate constants can be extracted from the batch experiments with enzymes entrapped in hydrogel, as discussed in section Effective Activity of Enzymes Entrapped in Hydrogels. The values of k_app, hydrogel, experimental_ are listed in Table 2 and are plotted in Figure 7A. On a first sight, the plot of the experimental apparent reaction rate constants shows the same qualitative distribution as the plot of the theoretical apparent reaction rate constants shown in Figure 5A. However, a closer look shows that the ratio between the smallest and highest values of the experimental apparent reaction rate constants is less pronounced. The reason for this is the stronger mass transfer limitation of enzymes with high activity when entrapped into the hydrogel (see section Comparison of Enzyme Activity in Solution and Entrapped in Hydrogel). Assuming a plug flow reactor with first order reaction kinetics, the conversion ratio Y can be calculated as follows:

(7) $$\begin{array}{l} Y=1-\frac{c_{substrate,out}}{c_{substrate,in}}\\ =1-exp\left\{\;\frac{-k_{app,hydrogel,experimental}\;*\;V_{hydrogel}}{\overset{\cdot }{V}}\right\} \end{array}$$

It is important to notice that because k_app, hydrogel, experimental_ refers to the reaction rate constant in the hydrogel, only the hydrogel volume must be considered in Equation (7). One, at first glance surprising, consequence of Equation (7) is that the volume of the open channels, which determine the residence time of the liquid in the reactor for a given flow rate, does not influence the conversion ratio. However, at a closer look this makes sense. The catalytic conversion only takes place within the hydrogel. Therefore, in case of a constant hydrogel volume a constant product formation rate is reached in the stationary phase, independent of the flow velocity of the fluid passing through the channels. The achieved conversion ratio is then determined by the product formation rate and the volume flow through the reactor.

The theoretical conversion yields calculated by Equation (7) as well as the experimentally achieved values are plotted in Figure 7B. From the plot it can be seen that the theoretical conversions yields are 84% in the case of β-Gal, and 78% in the case of ADH for the substrate (*S*)-HPP. In the case of BFD and the ADH-acetophenone system the theoretical conversion yield even approaches 100%.

A comparison with the experimentally achieved conversion yields shows that these are generally much smaller. The only exception is the ADH (*S*)-HPP system, where the experimental conversion yield is even slightly higher than the prediction. For the other enzymes the difference between the experimental and the theoretical conversion yield seems to strongly vary. However, one has to keep in mind that Equation (7) is not linear. For instance, the chosen reaction conditions in the reactor for the ADH-acetophenone system were optimal with respect to the achievement of high conversion yields. Therefore, even if the average volumetric activities in the hydrogel with entrapped ADH in the reactor would have been only a quarter of the theoretical value predicted from the batch experiments, it would be still sufficient for an almost complete conversion. In contrast, in the case of the reactor with entrapped β-Gal the conditions were less favorable, resulting in a theoretical conversion yield already limited to around 80%. Here, a reduction of the real average volumetric activity encountered in the reactor to about a quarter has a much stronger influence and reduces the achievable conversion yield to about the same extent. The question is, where the additional reduction of the average volumetric activity of the entrapped enzymes comes from. As the main reason, we assume it is due to the not ideally homogeneous flow patterns through the hydrogel inserts in the reactor. The printed hydrogels already show smaller channel structures in the fringe areas of the inserts, resulting from the printing process. This tendency might be increased when pushing the inserts in the reactor housing. Due to the strong influence of channel width onto the pressure drop, the fringe region of the inserts may experience a low or even no flow-through during the experiments. As a consequence, the mass transfer limitations in these fringe regions are strongly enhanced and they can contribute only very little to the productivity of the reactor. Only in the case of the ADH (*S*)-HPP system the apparent reaction rate constant of the entrapped enzyme seems to be so low that even increased distances through which mass transfer by diffusion has to take place, does not negatively influence the reactor performance. Nevertheless, at the current stage it cannot be fully explained, why this system does not show at least a small influence of the increased mass transfer limitation in the reactor.

## Conclusion

We presented a flexible, method for the immobilization of enzymes in hydrogel lattices under mild conditions based on 3D-printing. It can be used to estimate and optimize the performance of enzymes, regardless of their purity within continuous fixed-bed reactors. As immobilization is achieved by physical entrapment, the development of individual immobilization protocols can be omitted and the influence of protein sequence variations and reaction parameters onto enzyme activity can be screened directly. However, immobilization within the hydrogel matrix introduces mass transfer limitations which influence the volumetric activity of the biocatalyst compared to freely dissolved enzymes in solution. The resulting effectiveness factor decreases with increasing intrinsic enzyme activity. For four tested enzymatic reactions, the effectiveness factor calculated for the faster reactions was about 6–9%. It increased to 14% for the reduction of (*S*)-HPP by ADH, which is the slowest as well as the most complex of the investigated scenarios. Comparing calculated and experimental conversion in a flow reactor housing hydrogel lattices with entrapped enzyme, it can be stated that higher values of the effectiveness factor in batch experiments also hint at good performance in continuous flow. For the conversion of (*S*)-HPP by ADH the experimental conversion even surpassed the calculated one. Nevertheless, in general, the reaction rate of entrapped enzymes in the continuous flow system was lower than the corresponding reaction rates of entrapped enzymes in a batch reaction. Besides potential substrate and product adsorption within the reactor, non-optimal flow profiles decrease the yield of the continuous system. When the hydrogel lattices are 3D-printed, outer channels of the structures are smaller than the central ones due to the printing method (Schmieg et al., 2018). Upon placing the hydrogel lattices tight into the reactor housing, the deformation of the material will further decrease the size of the flow channels resulting in irregular flow patterns at the outer edges. To optimize the conversion, mechanically stable hydrogels with smaller strand diameters could minimize mass transfer limitations as well as improve the flow patterns. Furthermore, if the reactor cross-section is scaled up or the distance between the hydrogel strands is increased, the impact of boundary effects should be minimized.

Based on the data generated, the ideal reactor size and amount of enzyme for a given flow rate and almost complete conversion can be calculated. Manufacturing hydrogel immobilizates as well as reactor housings by rapid prototyping enables to set up enzymatic cascade reactions with matched, individual reactor sizes for different steps. By combining high-throughput batch screenings and such 3D-printed pilot plants the development of continuous processes can be accelerated and complex reactions can be optimized in a small scale.

## Author Contributions

MP and MF, the leaders of the respective research groups, contributed to the work by giving the idea for this project, helping in the case of scientific problems, and writing parts of the manuscript. BS was the scientist responsible for planning and performing experiments as well as writing the main part of the manuscript together with JD, who was responsible for providing theoretical and experimental knowledge on enzymatic process conditions. FK was responsible for the transfer and adaption of the HPLC setup from Jülich to Karlsruhe.

### Conflict of Interest Statement

The authors declare that the research was conducted in the absence of any commercial or financial relationships that could be construed as a potential conflict of interest.

## Abbreviations

ADH: Alcohol dehydrogenase from *Lactobacillus brevis*
BFD: Benzoylformate decarboxylase from *Pseudomonas putida*
β-Gal: β-Galactosidase from *Aspergillus oryzae*
ONPG: O-Nitrophenyl-β-d-galactopyranoside
PEG-DA: Poly(ethylene glycol) diacrylate
(*S*)-HPP: (*S*)-2-hydroxy-1-phenyl-propanone
(*S, S*)-PPD: (1*S*, 2*S*)-1-phenylpropane-1, 2-diol.

## References

[B1] Bettermann S. Schroeter B. Moritz H.-U. Pauer W. Fassbender M. Luinstra G. A. (2018). Continuous emulsion copolymerization processes at mild conditions in a 3D-printed tubular bended reactor. Chem. Eng. J. 338, 311–322. 10.1016/j.cej.2018.01.038

[B2] Brakowski R. Pontius K. Franzreb M. (2016). Investigation of the transglycosylation potential of ß-Galactosidase from *Aspergillus oryzae* in the presence of the ionic liquid [Bmim][PF6]. J. Mol. Catal. B Enzym. 130, 48–57. 10.1016/j.molcatb.2016.05.006

[B3] Cao L. (2005). Carrier-Bound Immobilized Enzymes: Principles, Applications and Design. Weinheim: Wiley-VCH 10.1002/3527607668

[B4] Deichmann U. Schuster S. Mazat J.-P. Cornish-Bowden A. (2014). Commemorating the 1913 Michaelis–Menten paper “Die Kinetik der Invertinwirkung”: three perspectives. FEBS J. 281, 435–463. 10.1111/febs.12598 24180270

[B5] Deng K. Guenther J. M. Gao J. Bowen B. P. Tran H. Reyes-Ortiz V.. (2015). Development of a high throughput platform for screening glycoside hydrolases based on oxime-NIMS. Front. Bioeng. Biotechnol. 3:153. 10.3389/fbioe.2015.00153 26528471 PMC4603251

[B6] DiCosimo R. McAuliffe J. Poulose A. J. Bohlmann G. (2013). Industrial use of immobilized enzymes. Chem. Soc. Rev. 42, 6437–6474. 10.1039/c3cs35506c 23436023

[B7] Döbber J. Gerlach T. Offermann H. Rother D. Pohl M. (2018a). Closing the gap for efficient immobilization of biocatalysts in continuous processes: HaloTag^TM^ fusion enzymes for a continuous enzymatic cascade towards a vicinal chiral diol. Green Chem. 20, 544–552. 10.1039/C7GC03225K

[B8] Döbber J. Pohl M. (2017). HaloTag^TM^: evaluation of a covalent one-step immobilization for biocatalysis. J. Biotechnol. 241, 170–174. 10.1016/j.jbiotec.2016.12.004 27923737

[B9] Döbber J. Pohl M. Ley S. V. Musio B. (2018b). Rapid, selective and stable HaloTag-LbADH immobilization directly from crude cell extract for the continuous biocatalytic production of chiral alcohols and epoxides. React. Chem. Eng. 3, 8–12. 10.1039/C7RE00173H

[B10] England C. G. Luo H. Cai W. (2015). HaloTag technology: a versatile platform for biomedical applications. Bioconjug. Chem. 26, 975–986. 10.1021/acs.bioconjchem.5b00191 25974629 PMC4482335

[B11] Fraas R. Diehm J. Franzreb M. (2017). Automated solid-phase protein modification with integrated enzymatic digest for reaction validation: application of a compartmented microfluidic reactor for rapid optimization and analysis of protein biotinylation. Front. Bioeng. Biotechnol. 5:72. 10.3389/fbioe.2017.00072 29181376 PMC5693853

[B12] Gelhausen M. G. Feuerbach T. Schubert A. Agar D. W. (2018). 3D printing for chemical process laboratories I: materials and connection principles. Chem. Eng. Technol. 41, 618–627. 10.1002/ceat.201700294

[B13] Gocke D. Graf T. Brosi H. Frindi-Wosch I. Walter L. Muller M. (2009). Comparative characterisation of thiamin diphosphate-dependent decarboxylases. J. Mol. Catal. B-Enzymatic 61, 30–35. 10.1016/j.molcatb.2009.03.019

[B14] Grosová Z. Rosenberg M. Rebroš M. (2008). Perspectives and applications of immobilised β-galactosidase in food industry - A review. Czech J. Food Sci. 26, 1–14. 10.17221/1134-CJFS

[B15] Gutmann B. Kockinger M. Glotz G. Ciaglia T. Slama E. Zadravec M. (2017). Design and 3D printing of a stainless steel reactor for continuous difluoromethylations using fluoroform. React. Chem. Eng. 2, 919–927. 10.1039/C7RE00176B

[B16] Hasson M. S. Muscate A. McLeish M. J. Polovnikova L. S. Gerlt J. A. Kenyon G. L. (1998). The crystal structure of benzoylformate decarboxylase at 1.6 angstrom resolution: diversity of catalytic residues in thiamin diphosphate-dependent enzymes. Biochemistry 37, 9918–9930. 10.1021/bi973047e 9665697

[B17] Iding H. Dunnwald T. Greiner L. Liese A. Muller M. Siegert P.. (2000). Benzoylformate decarboxylase from *Pseudomonas putida* as stable catalyst for the synthesis of chiral 2-hydroxy ketones. Chem. Eur. J. 6, 1483–1495. 10.1002/(SICI)1521-3765(20000417)6:8<1483::AID-CHEM1483>3.0.CO;2-S 10840971

[B18] Kamble M. P. Yadav G. D. (2017). Kinetic resolution of (R,S)-alpha-tetralol by immobilized candida antarctica lipase B: comparison of packed-bed over stirred-tank batch bioreactor. Ind. Eng. Chem. Res. 56, 1750–1757. 10.1021/acs.iecr.6b03401

[B19] Kazenwadel F. Biegert E. Wohlgemuth J. Wagner H. Franzreb M. (2016). A 3D-printed modular reactor setup including temperature and pH control for the compartmentalized implementation of enzyme cascades. Eng. Life Sci. 16, 560–567. 10.1002/elsc.201600007

[B20] Kihumbu D. Stillger T. Hummel W. Liese A. (2002). Enzymatic synthesis of all stereoisomers of 1-phenylpropane-1,2-diol. Tetrahedr. Asymmetry 13, 1069–1072. 10.1016/S0957-4166(02)00247-1

[B21] Kulig J. Simon R. C. Rose C. A. Husain S. M. Hackh M. Ludeke S. (2012). Stereoselective synthesis of bulky 1,2-diols with alcohol dehydrogenases. Catal. Sci. Technol. 2, 1580–1589. 10.1039/c2cy20120h

[B22] Kulishova L. Dimoula K. Jordan M. Wirtz A. Hofmann D. Santiago-Schubel B. (2010). Factors influencing the operational stability of NADPH-dependent alcohol dehydrogenase and an NADH-dependent variant thereof in gas/solid reactors. J. Mol. Catal. B-Enzymatic 67, 271–283. 10.1016/j.molcatb.2010.09.005

[B23] Laidler K. J. Velick S. F. (1983). Symbolism and terminology in enzyme kinetics: recommendations 1981 (Nomenclature Committee of the International Union of Biochemistry). Arch. Biochem. Biophys. 224, 732–740. 10.1016/0003-9861(83)90262-X 6870287

[B24] Leuchs S. Greiner L. (2011). Alcohol dehydrogenase from *Lactobacillus brevis*: a versatile robust catalyst for enantioselective transformations. Chem. Biochem. Eng. Q. 25, 267–281. Available online at: https://hrcak.srce.hr/69863

[B25] Maksimainen M. M. Lampio A. Mertanen M. Turunen O. Rouvinen J. (2013). The crystal structure of acidic β-galactosidase from *Aspergillus oryzae*. Int. J. Biol. Macromol. 60, 109–115. 10.1016/j.ijbiomac.2013.05.003 23688418

[B26] Miller J. H. (1972). Experiments in Molecular Genetics - E. coli. University of Michigan; Cold Spring Harbor Laboratory, NY.

[B27] Mlichova Z. Rosenberg M. (2006). Current trends of beta-galactosidase application in food technology. J. Food Nutr. Res. 45, 47–54. Available online at: http://www.vup.sk/en/index.php?mainID=2&navID=34&version=2&volume=45&article=783

[B28] Mohamad N. R. Marzuki N. H. C. Buang N. A. Huyop F. Wahab R. A. (2015). An overview of technologies for immobilization of enzymes and surface analysis techniques for immobilized enzymes. Biotechnol. Biotechnol. Equip. 29, 205–220. 10.1080/13102818.2015.1008192 26019635 PMC4434042

[B29] Niefind K. Muller J. Riebel B. Hummel W. Schomburg D. (2003). The crystal structure of R-specific alcohol dehydrogenase from *Lactobacillus brevis* suggests the structural basis of its metal dependency. J. Mol. Biol. 327, 317–328. 10.1016/S0022-2836(03)00081-0 12628239

[B30] Peris E. Okafor O. Kulcinskaja E. Goodridge R. Luis S. V. Garcia-Verdugo E. (2017). Tuneable 3D printed bioreactors for transaminations under continuous-flow. Green Chem. 19, 5345–5349. 10.1039/C7GC02421E

[B31] Porta R. Benaglia M. Puglisi A. (2016). Flow chemistry: recent developments in the synthesis of pharmaceutical products. Org. Process Res. Dev. 20, 2–25. 10.1021/acs.oprd.5b00325

[B32] Radtke C. P. Hillebrandt N. Hubbuch J. (2017). The biomaker: an entry-level bioprinting device for biotechnological applications. J. Chem. Technol. Biotechnol. 93, 792–799. 10.1002/jctb.5429

[B33] Rao Z. X. Patel B. Monaco A. Cao Z. J. Barniol-Xicota M. Pichon E. (2017). 3D-printed polypropylene continuous-flow column reactors: exploration of reactor utility in SNAr reactions and the synthesis of bicyclic and tetracyclic heterocycles. Eur. J. Organ. Chem. 2017, 6499–6504. 10.1002/ejoc.201701111

[B34] Riebel B. (1996). Biochemische und Molekularbiologische Charakterisierung neuer Mikrobieller NAD(P)-Abhängiger Alkoholdehydrogenasen. Doctoral thesis, Heinrich-Heine-Universität, Düsseldorf.

[B35] Robinson P. K. (2015). Enzymes: principles and biotechnological applications. Essays Biochem. 59, 1–41. 10.1042/bse0590001 26504249 PMC4692135

[B36] Rodríguez C. Borzecka W. Sattler J. H. Kroutil W. Lavandera I. Gotor V. (2014). Steric vs. electronic effects in the Lactobacillus brevis ADH-catalyzed bioreduction of ketones. Organ. Biomol. Chem. 12, 673–681. 10.1039/C3OB42057D 24302226

[B37] Schlieben N. H. Niefind K. Muller J. Riebel B. Hummel W. Schomburg D. (2005). Atomic resolution structures of R-specific alcohol dehydrogenase from *Lactobacillus brevis* provide the structural bases of its substrate and cosubstrate specificity. J. Mol. Biol. 349, 801–813. 10.1016/j.jmb.2005.04.029 15896805

[B38] Schmieg B. Schimek A. Franzreb M. (2018). Development and performance of a 3D-printable poly(ethylene glycol) diacrylate hydrogel suitable for enzyme entrapment and long-term biocatalytic applications. Eng. Life Sci. 18, 659–667. 10.1002/elsc.201800030 PMC6999379 32624946

[B39] Sheldon R. A. (2007). Enzyme immobilization: the quest for optimum performance. Adv. Synth. Catal. 349, 1289–1307. 10.1002/adsc.200700082

[B40] Sheldon R. A. Woodley J. M. (2018). Role of biocatalysis in sustainable chemistry. Chem. Rev. 118, 801–838. 10.1021/acs.chemrev.7b00203 28876904

[B41] Tanaka Y. Kagamiishi A. Kiuchi A. Horiuchi T. (1975). Purification and propoerties of beta-galactosidase from *Aspergillus oryzae*. J. Biochem. 77, 241–247. 236999

[B42] Tufvesson P. Lima-Ramos J. Nordblad M. Woodley J. M. (2011). Guidelines and cost analysis for catalyst production in biocatalytic processes. Organ. Process Res. Dev. 15, 266–274. 10.1021/op1002165

[B43] Valinger D. Prescki A. V. Kurtanjek Z. Pohl M. Blazevic Z. F. Vasic-Racki D. (2014). Continuous enzymatic carboligation of benzaldehyde and acetaldehyde in an enzyme ultrafiltration membrane reactor and laminar flow microreactors. J. Mol. Catal. B-Enzymatic 102, 132–137. 10.1016/j.molcatb.2014.02.003

[B44] Wachtmeister J. Jakoblinnert A. Rother D. (2016). Stereoselective two-step biocatalysis in organic solvent: toward all stereoisomers of a 1,2-diol at high product concentrations. Org. Process Res. Dev. 20, 1744–1753. 10.1021/acs.oprd.6b00232

[B45] Weiser D. Nagy F. Banoczi G. Olah M. Farkas A. Szilagyi A. (2017). Immobilization engineering - how to design advanced sol-gel systems for biocatalysis? Green Chem. 19, 3927–3937. 10.1039/C7GC00896A

[B46] Wilcocks R. Ward O. P. (1992). Factors affecting 2-hydroxypropiophenone formation by benzoylformate decarboxylase from *Pseudomonas putida*. Biotechnol. Bioeng. 39, 1058–1063. 10.1002/bit.260391010 18600905

[B47] Zhang Z. Zhang R. Chen L. McClements D. J. (2016). Encapsulation of lactase (β-galactosidase) into κ-carrageenan-based hydrogel beads: impact of environmental conditions on enzyme activity. Food Chem. 200, 69–75. 10.1016/j.foodchem.2016.01.014 26830562

